# Novel Resonance-Based Wireless Power Transfer Using Mixed Coupling

**DOI:** 10.3390/s20247277

**Published:** 2020-12-18

**Authors:** SangWook Park, Seungyoung Ahn

**Affiliations:** 1 College of Information and Communication Engineering, Daegu University, Gyeongsan 38453, Korea; 2 The Cho Chun Shik Graduate School for Green Transportation, Korea Advanced Institute of Science and Technology, Daejeon 34141, Korea; sahn@kaist.ac.kr

**Keywords:** electric coupling, magnetic coupling, mixed coupling, mode decomposition, near field communication, wireless power transfer

## Abstract

This study presents an equivalent circuit model for the analysis of wireless power transfer (WPT) through both electric and magnetic couplings using merely a resonant coupler. Moreover, the frequency split phenomenon, which occurs when transmitting couplers are near receiving couplers, is explained. This phenomenon was analyzed using simple circuit models derived via a mode decomposition technique. To verify the proposed method, a resonant coupler using mixed coupling was designed and its efficiency was compared with the result obtained using a commercial electromagnetic solver. The results of this study are expected to aid in designing various WPT couplers or sensor antennas by selecting electric, magnetic, or mixed couplings. Furthermore, the results of this study are expected to be applied to technologies that sense objects, or simultaneously transmit and receive information and power wirelessly.

## 1. Introduction

The resonance-based wireless power transfer (WPT) technique using strong magnetic coupling was proposed by Kurs et al. in [1]. Since then, many scholars and researchers have been conducting research on the WPT. In recent years, simultaneous implementation of the WPT and wireless communication was studied by combining near field communication (NFC) and radio frequency identification (RFID) technologies. The reason behind is because the role of antenna or coupler in the WPT and wireless communications is not different in terms of sending and receiving electromagnetic energy. Finally, in the WPT and wireless communication technologies, the three methods of exchanging electromagnetic energy can be largely divided into magnetic field coupling, electric field coupling, and a method to employ both couplings. In [1], it was concluded that the efficiency of the WPT is proportional to the product of the coupling coefficient and quality factor. This coupling can be implemented via a mutual magnetic field between two loops, i.e., inductive coupling, and/or a mutual electric field between the two conductor plates, i.e., capacitive coupling. Further, the power is transferred primarily through inductive coupling. In this study, inductive wireless power transfer (IPT) refers to the magnetic field coupling, capacitive wireless power transfer (CPT) refers to the electric field coupling, and mixed coupling wireless power transfer (MPT) refers to both the magnetic and electric field couplings. Lately, research on the CPT method is on the rise [2,3], and very few studies on the MPT method have been conducted [4,5]. In [4], WPT is implemented via combined inductive coupling and capacitive coupling. In [5], inductive coupling and capacitive coupling are integrated into one coupler for WPT. However, their proposed coupler structure was very large and complicated, making it complex for practical application.

In [6], IPT and CPT have demonstrated different electrical characteristics due to different main couplings; therefore, it is necessary to design a more suitable coupling method according to the application target to maximize the benefits that can be derived from the electrical characteristics of each method. NFC uses a magnetic field coupling having a resonance frequency of 13.56 MHz [7,8,9,10,11,12,13,14]. In the resonance-based IPT using 6.78 MHz as the operating frequency, the harmonic component causes the problem of electromagnetic interference to NFC [15,16,17]. We can solve the problem of electromagnetic interference with NFC by using the electric coupling method, such as CPT. As such, it is significant to design antennas, such as NFC and RFID, for communication or couplers for WPT in various ways according to the situation and purpose [18,19,20,21,22,23,24].

In the WPT method using magnetic field coupling, if there is a metal object between the transmitter and the receiver, a heating issue occurs due to the current induced in the metal object. For example, gum wrapper may lead to a fire. In addition, if living organisms are placed between the transmitter and the receivers, exposure to electromagnetic fields can cause adverse health effects. These problems are even more serious when demanding high power, such as an electric vehicle. Therefore, there is a need for technology to detect or sense foreign objects or living objects [25,26,27,28,29,30,31]. For detection, a small power electromagnetic field can be generated to sense metal or living objects. Metal objects can be sensed using magnetic resonance, and living objects are dielectrics, so they can be sensed using electric field resonance.

According to the above statements, it is necessary to have an in-depth understanding of IPT, CPT and MPT to be able to apply a technique that solves interference problems, or a technique that transfers power and information simultaneously. Therefore, this study aimed to establish an equivalent circuit analysis for designing IPT, CPT, and MPT. In designing WPT and wireless communication systems, the antenna or coupler part can be expressed by a simple equivalent circuit composed of the lumped parameters R, L and C. The reason behind is that analyzing an equivalent circuit without dealing with a complex electromagnetic theory is much easier. Therefore, the equivalent circuit analysis method is significantly important. If the two-port network equivalent circuit representing the transmitting and receiving antennas or couplers can be obtained, the following interpretations can be readily performed in designing the system: (1) The transmission and reflection efficiencies can be easily interpreted by switching directly from the two-port network expression to the scattering matrix; (2) By applying terminal conditions to both ports, the transmission and reflection efficiencies in the actual circuit can be analyzed; (3) The matching circuit can be easily calculated using two-port network expression; and (4) Predicting the results of various situations, such as changes in the transmission distance, will be straightforward.

Consequently, it is vital to establish an equivalent circuit and extract the parameters to express the antenna or coupler part. The most uncomplicated method of extracting equivalent circuit parameters is using an RLC meter. The higher the frequency band, the more difficult it becomes to use the RLC meter. Therefore, in the higher frequency band, a common method is to measure S-parameters using a vector network analyzer (VNA) and convert them back into the input impedance. The study primarily deals with the establishment of an appropriate equivalent circuit with extracted equivalent RLC parameters and its analysis by using the electromagnetic analysis tools in place of the measurements to obtain S-parameters. In this paper, we propose an equivalent circuit model for MPT that uses both magnetic field coupling and electric field coupling, i.e., mixed coupling. This model includes an analysis of IPT and CPT. This paper also presents an intuitive analysis by explaining it as a simple circuit model for two resonant modes, which are generated when the two couplers are strongly coupled, using mode decomposition techniques. Finally, to verify our proposed equivalent circuit model, the efficiency of our model is compared with that of a commercial electromagnetic solver with a simple example for MPT.

The major contributions of this study are as follows:

1. Established the MPT equivalent circuit model;
2. Proposed a method to interpret the MPT equivalent circuit model using mode decomposition technique;
3. Proposed a basic and compact MPT structure based on the intuitive insight.

## 2. Equivalent Circuit Model for Wireless Power Transfer Using Mixed Coupling

**Figure 1:**
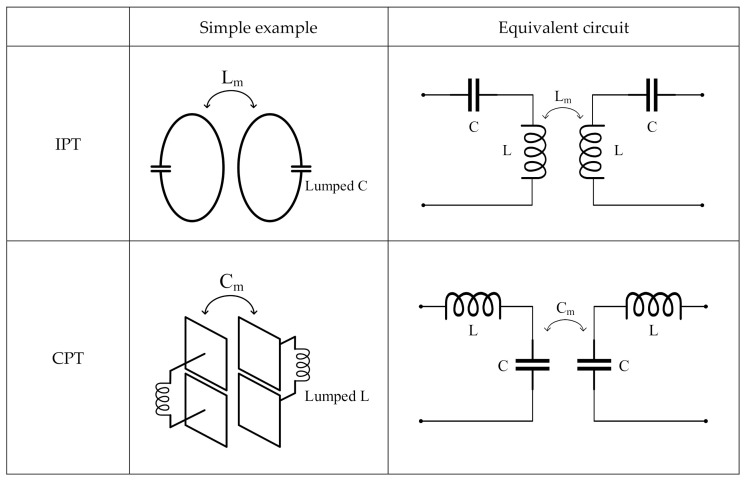

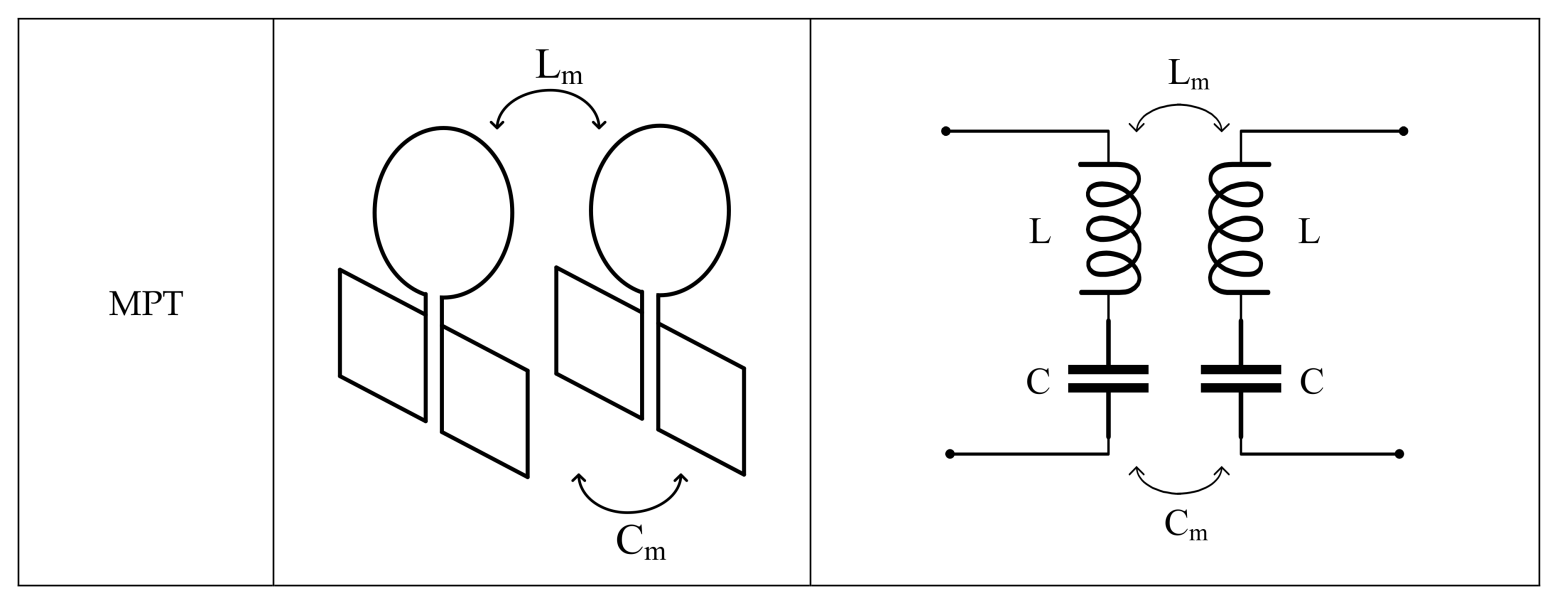
Structural examples of wireless power transfer (WPT) using magnetic coupling, electric coupling, and mixed coupling and their equivalent circuits.

Figure 1 shows simple examples and the corresponding equivalent circuits for IPT, CPT, and MPT. In the example of IPT, magnetic field energy is transmitted by Faraday’s law by a coil structure (magnetic coupling), and the desired resonant frequency can be determined by adding lumped C. In the example of CPT, electric field energy is transferred by charge induction between parallel conductor plates (electric coupling), and the desired resonance frequency can be determined by adding a lumped L. In the example of MPT, the coil structure and the parallel plate structure transmit electromagnetic energy by generating magnetic and electric field coupling, respectively, and resonate by the L and C of the coil and the conductor plate itself without adding additional lumped elements. We can easily obtain impedance parameters (Z-parameters) or admittance parameters (Y-parameters) from each equivalent circuit. Then, scattering parameters (S-parameters) can be obtained from the interrelation of parameters, and the characteristics of the efficiency for each structure can be obtained using S21; that is, the transmission coefficient. Because the Z-parameters circuit for magnetic coupling obtained using Equation (1) and the Y-parameters circuit for electric coupling using Equation (2) are connected in series in the structure of MPT, the equivalent Z-parameters of MPT can be expressed as Equation (3).

(1) $${\left[Z\right]}_{IPT}=\left[\begin{array}{cc} j\omega L & j\omega L_{m}\\ j\omega L_{m} & j\omega L \end{array}\right]$$

(2) $${\left[Y\right]}_{CPT}=\left[\begin{array}{cc} j\omega C & j\omega C_{m}\\ j\omega C_{m} & j\omega C \end{array}\right]$$

(3) $${\left[Z\right]}_{MPT}={\left[Z\right]}_{IPT}+{\left[Y\right]}_{CPT}^{-1}$$

## 3. Equivalent Circuit Analysis with Mode Decomposition

**Figure 2:**
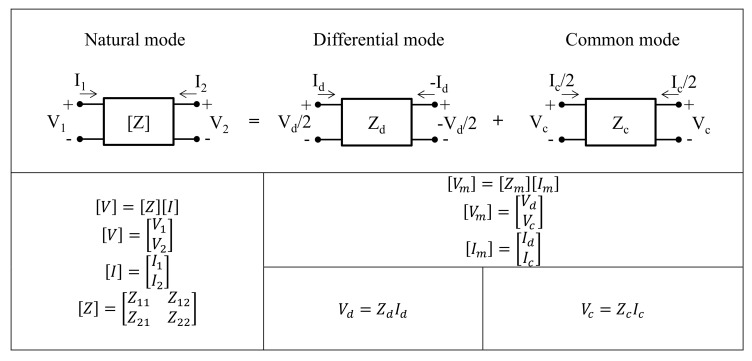
Two-port network expression of natural mode with differential and common modes.

In this section, we address the frequency split phenomenon, which occurs when the coupling coefficient between transmitter and receiver is large, with equivalent circuit analysis using the mode decomposition technique [32]. The natural mode of two-port networks can be divided into two representative orthogonal modes, namely differential mode (DM) and common mode (CM), by the mode decomposition technique, as shown in Figure 2. Natural voltage and current matrices can be divided into mode voltage and current matrices using mode conversion matrices, $T_{v}$ and $T_{i}$, as shown below:

(4) $$\left[V\right]=T_{v}\left[V_{m}\right],\;\;T_{v}=\left[\begin{array}{cc} \frac{1}{2} & 1\\ -\frac{1}{2} & 1 \end{array}\right]$$

(5) $$\left[I\right]=T_{i}\left[I_{m}\right],\;\;T_{i}=\left[\begin{array}{cc} 1 & \frac{1}{2}\\ -1 & \frac{1}{2} \end{array}\right]$$

From the above relations, the mode impedance parameters, $\left[Z_{m}\right]$ are expressed as

(6) $$\left[Z_{m}\right]=T_{v}\left[Z\right]T_{i}^{-1}=\left[\begin{array}{cc} Z_{11}+Z_{22}-Z_{12}-Z_{21} & \frac{1}{2}\left(Z_{11}+Z_{12}-Z_{21}-Z_{22}\right)\\ \frac{1}{2}\left(Z_{11}+Z_{21}-Z_{12}-Z_{22}\right) & \frac{1}{4}\left(Z_{11}+Z_{22}+Z_{12}+Z_{21}\right) \end{array}\right]$$

If the coupler is symmetric ($Z_{11}=Z_{22}$) and reciprocal ($Z_{12}=Z_{21}$), $\left[Z_{m}\right]$ can be simplified as follows:

(7) $$\left[Z_{m}\right]=\left[\begin{array}{cc} 2(Z_{11}-Z_{12}) & 0\\ 0 & \frac{1}{2}\left(Z_{11}+Z_{22}\right) \end{array}\right]=\left[\begin{array}{cc} Z_{d} & 0\\ 0 & Z_{c} \end{array}\right]$$

This indicates that if we know the impedance of each mode, the natural impedance matrices can be reconstructed from Equation (6).

**Figure 3:**
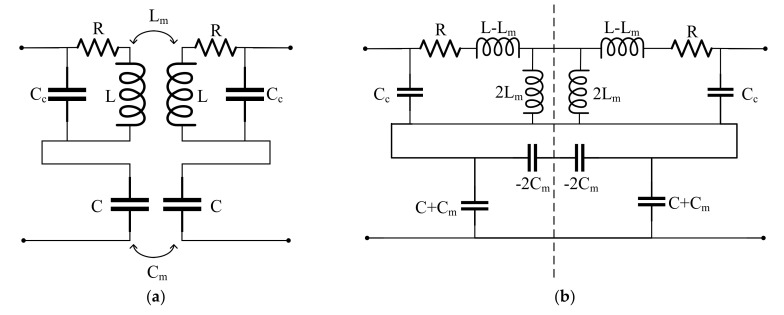

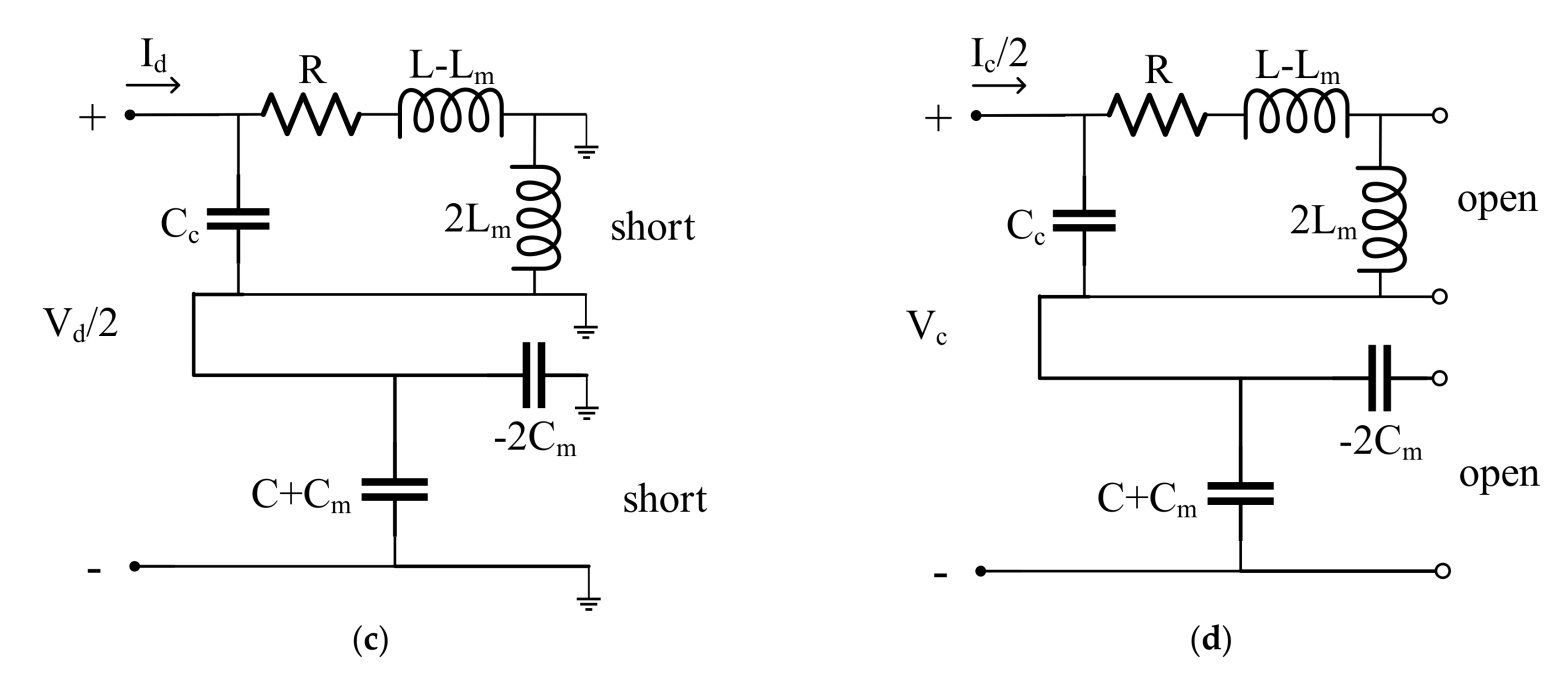
(**a**) Practical equivalent circuit for practical mixed coupling wireless power transfer (MPT). (**b**) An alternative form of the equivalent circuit with pi and T networks. Simplified equivalent circuit in the case of (**c**) differential mode and (**d**) common mode.

Next, the mechanism of frequency split is described with the practical equivalent circuit model of MPT by using the mode decomposition technique. The practical equivalent model of MPT can be expressed as in Figure 3a. Figure 1 demonstrates the equivalent circuit model for the MPT, which is a simple model to enhance the understanding of the IPT, CPT and MPT. Whereas the equivalent circuit model of the MPT in Figure 3a is more realistic and precise considering resistance R and parasitic capacitor C_c_. In Section 4, we will discuss the predictability of the practical equivalent model compared to the simplified equivalent model. In the coil-shaped portion for magnetic field coupling, a parasitic capacitance, C_c_, exists between the wires of the coil and the self-inductance, L. Additionally, the self-capacitance, C, exists between the metal bodies of the plate-type structure for the electric field coupling, and the total loss resistance of the MPT is represented by R. The mutual inductance, L_m_, and mutual capacitance, C_m_, refer to the magnetic and electrical coupling between the transmitter and receiver. The equivalent circuit of MPT in Figure 3a can be expressed as shown in Figure 3b by the pi and T equivalent circuits. In the middle (the symmetrical plane indicated by the dashed line) of the MPT equivalent circuit, DM can be regarded as a short circuit (an electric wall) as shown in Figure 3c, and CM can be regarded as an open circuit (a magnetic wall) as shown in Figure 3d. Thus, the impedance and resonance frequency of each mode are expressed as

(8) $$Z_{d}=2\left[\left\{\frac{1}{j\omega C_{c}}//R+j\omega \left(L-L_{m}\right)\right\}+\frac{1}{j\omega \left(C-C_{m}\right)}\right]$$

(9) $$Z_{c}=\frac{1}{2}\left[\left\{\frac{1}{j\omega C_{c}}//R+j\omega \left(L+L_{m}\right)\right\}+\frac{1}{j\omega \left(C+C_{m}\right)}\right]$$

(10) $$f_{d}=\frac{1}{2\pi \sqrt{(\left(L-L_{m}\right)\left(C-C_{m}+C_{c}\right)}}$$

(11) $$f_{c}=\frac{1}{2\pi \sqrt{(\left(L+L_{m}\right)\left(C+C_{m}+C_{c}\right)}}$$

The mode decomposition analysis helps understand that the frequency split phenomenon is caused by mutual inductance and mutual capacitance. The coupling effect reduces the charge-storing capability and the stored flux in the single resonator when the electric wall is inserted in the symmetrical plane of the coupled structure (DM case). In contrast, the coupling effect enhances these properties when the magnetic wall is inserted in the symmetric plane (CM case). From the equivalent parameters, the coupling coefficient of the MPT, *k_em_* can be expressed as

(12) $$k_{em}=\frac{CL_{m}+LC_{m}}{LC+L_{m}C_{m}}=\frac{k_{e}+k_{m}}{1+k_{e}k_{m}}$$

where

(13) $$k_{e}=\frac{C_{m}}{C},\;k_{m}=\frac{L_{m}}{L}$$

If $k_{e}k_{m}\ll 1$, then Equation (12) is expressed as

(14) $$k_{em}\approx k_{e}+k_{m}$$

## 4. Results and Discussion

**Figure 4:**
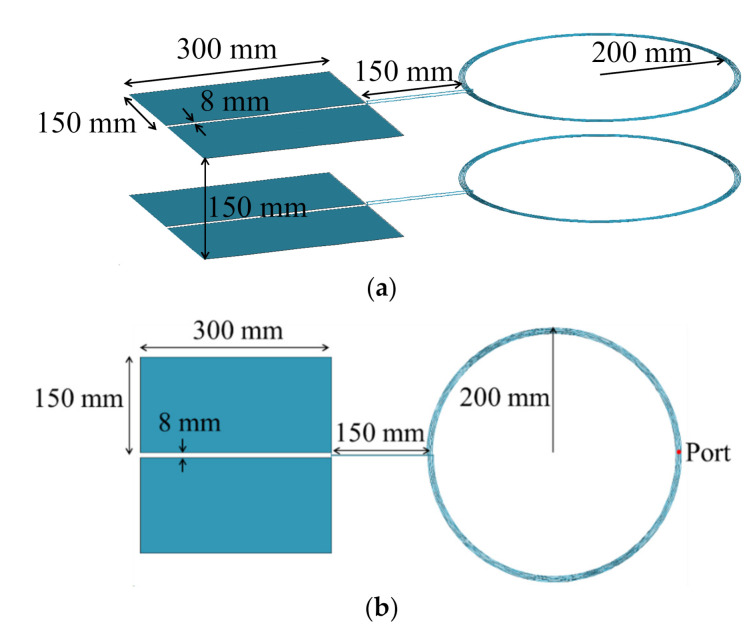
Proposed MPT structure: (**a**) bird view and (**b**) top view.

**Figure 5:**
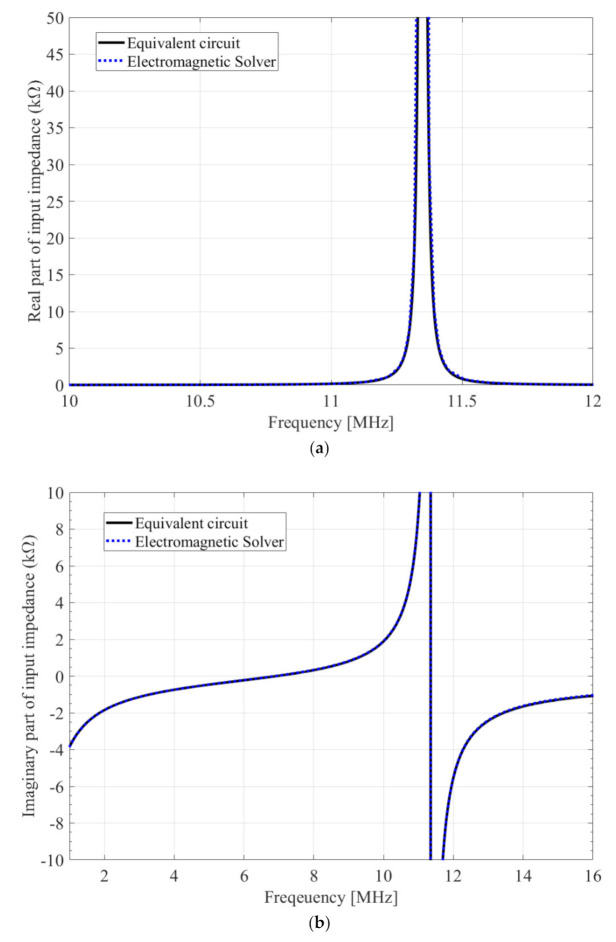
Input impedance by electromagnetic solver (FEKO) simulation and extracted equivalent circuit parameters (RLC): (**a**) real part; (**b**) imaginary part.

For the verification, we propose an example of the MPT structure and discuss the results obtained through an electromagnetic solver (FEKO) simulation, which is a commercial tool for electromagnetic analysis based on the method of moments [33] and the equivalent circuit model. The proposed MPT structure is shown in Figure 4. The basic structure is the same as that of the MPT in Figure 1; however, the coil has five turns. The coupler exhibits a coil structure and a plate structure, in which the magnetic field and electric field are mainly coupled, respectively. The equivalent circuit parameters (RLC) [34] were extracted using the MPT structure by comparing the real and imaginary parts of the input impedance by FEKO simulation as shown in Figure 5. In addition, the resonant frequencies and coupling coefficients were obtained using the extracted parameters and listed in Table 1. As shown in Figure 5a, the real part of the input impedance and extracted equivalent parameter, R, are well matched. Further, Figure 5b confirms that the imaginary part of the input impedance and extracted equivalent parameters C, L and C_c_ are matched appropriately. The actual input impedance of one coupler of the MPT is as follows.

(15) $$Z=\left(R+j\omega L\right)\;//\;\frac{1}{j\omega C_{c}}+\frac{1}{j\omega C}$$

The real and imaginary parts of this input impedance can be graphed as shown in Figure 5. According to (15), if the resistance R is neglected, the series and parallel resonance frequencies will be respectively derived as follows.

(16) $$\mathrm{Series}\;\mathrm{resonance}\;\mathrm{frequency}\;f_{series}=\frac{1}{2\pi \sqrt{L\left(C_{c}+C\right)}}$$

(17) $$\mathrm{Parallel}\;\mathrm{resonance}\;\mathrm{frequency}\;f_{parallel}=\frac{1}{2\pi \sqrt{LC_{c}}}$$

As presented in Table 1, if we substitute the extracted L, C and C_c_ into the resonance frequency formulas, the series and parallel resonance frequencies will become 6.93 MHz and 11.35 MHz, respectively. It can be confirmed that the two resonance frequencies can be seen in the imaginary part of the graph in Figure 5b. C and L can be extracted from the front (band lower than series resonance frequency) and middle parts of the imaginary part, respectively, when the imaginary part of the input impedance is obtained using an electromagnetic solver or the VNA. Further, C_c_ can be extracted from the parallel resonance frequency. When L, C and C_c_ are extracted, the remaining R can be obtained from the real part of the input impedance. In the proposed MPT structure shown in Figure 4, L_m_ and C_m_ can be obtained separately by merely simulating the coil and the plate-type structure, respectively.

**Figure 6:**
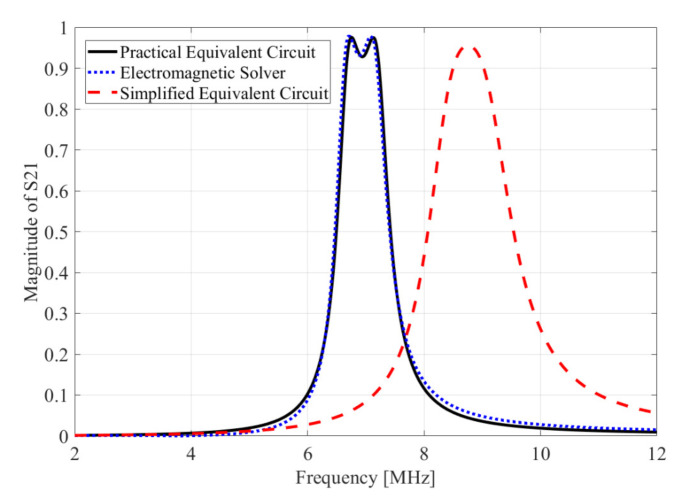
S21 results obtained by the electromagnetic solver (FEKO), practical equivalent circuit and simplified equivalent circuit.

Figure 6 shows that the graph of S21 calculated by electromagnetic solver (FEKO) agrees well with the results obtained from the practical equivalent circuit model (see Figure 3a). However, the simplified equivalent circuit model (see Figure 1) is indicated by the red dashed line in Figure 6. It presents completely different results compared to the electromagnetic solver and the practical equivalent circuit model. Therefore, our proposed method can accurately predict the electric coupling, magnetic coupling or mixed coupling.

## 5. Conclusions

In this paper, we proposed an equivalent circuit model of MPT and presented an analysis by implementing the mode decomposition technique along with the description of the frequency split phenomenon. Unlike IPT and CPT, a separate lumped C or L was unnecessary for the MPT structure because the coupler itself resonates. The exposure level of the undesirable external electric and magnetic fields can be varied by appropriately designing the electric and magnetic couplings. The results of this research are expected facilitate the design of the couplers of various WPT methods and antennas, such as NFC and RFID, in the future. As further work, we will study a simultaneous wireless information and power transfer system using the proposed MPT method and apply it to various sensors with continuous power.

## Figures and Tables

**Table 1:** Equivalent Parameters of MPT.

R	0.5 Ω	f_d_	7.21 MHz
L	8.137 µH	f_c_	6.67 MHz
C	40.73 pF	f_0_	6.93 MHz
C_c_	24.17 pF	K_em_	0.086
L_m_	0.529 µH	K_e_	0.021
C_m_	0.866 pF	K_m_	0.065
